# Sex-Specific Association between Environmental Tobacco Smoke Exposure and Asthma Severity among Adults with Current Asthma

**DOI:** 10.3390/ijerph19095036

**Published:** 2022-04-21

**Authors:** Benjamin J. Becerra, Devin Arias, Monideepa B. Becerra

**Affiliations:** 1 Center for Health Equity, Department of Information and Decision Sciences, California State University-San Bernardino, San Bernardino, CA 92407, USA; benjamin.becerra@csusb.edu; 2 Center for Health Equity, Department of Health Science & Human Ecology, California State University-San Bernardino, San Bernardino, CA 92407, USA; devin.arias@csusb.edu

**Keywords:** asthma, tobacco, firsthand smoke, secondhand smoke, environmental tobacco smoke, biological sex

## Abstract

Background: Tobacco smoke has been associated with negative health outcomes, including those with chronic respiratory illnesses, such as asthma. This study aimed to assess the relationship between exposure to environmental tobacco smoke (ETS), as well as tobacco use (cigarette and electronic cigarettes), on asthma severity among adults with current asthma, with stratification by sex to understand potential biological sex differences. Methods: The study population consisted of Californian adults 18 years or older with self-reported physician/health care diagnosis of asthma and still having current asthma from 2020 California Health Interview Survey. All descriptive statistics and analyses were sex-stratified and survey-weighted. Crosstabulations were used to understand the association between asthma attack and ETS or firsthand smoke exposure, while binary logistic regression models were used to assess the effect of ETS exposure, current smoking status, and control variables on asthma attack in the past 12 months, with a sub-analysis among non-smoking adults with asthma. Results: Among the primary variable of interest, 35% of males and 30% of females reported ETS exposure in the past 12 months, while 13% of males and 6% of females reported being a current smoker. Past year asthma attack was reported among 43% and 55% of males and females, respectively. Among males, after adjusting for all control variables, asthma attack was significantly higher among those with ETS exposure (OR: 1.75, 95% CI: 1.01–3.02) and among current smokers (OR: 3.82, 95% CI: 1.49, 9.81). Male non-smokers with ETS exposure had a 109% higher odds of asthma attack, compared to non-exposure individuals. Conclusion: Using a population-based survey, our results highlight the ongoing burden of tobacco use and exposure particularly among males with current asthma, further corroborate the literature on the relationship between tobacco and asthma, and highlight putative sex-specific outcomes.

## 1. Background

Tobacco use remains a leading cause of preventable mortality, with 7 million annual global deaths attributable to direct use [1]. Further, smoking tobacco not only impacts the user, but also impacts nonsmokers due to secondhand smoke exposure (also known as environmental tobacco smoke (ETS)), with 1.2 million global tobacco deaths attributable to such exposure [1]. In addition, emergent studies on thirdhand smoke have shown harmful biological outcomes resulting from residual nicotine and chemical components that remain in residential settings shared or previously inhabited by smokers [2,3].

Furthermore, in the United States, 16 million people are estimated to be living with an illness resulting from tobacco use, with 480,000 annual deaths attributed to cigarette smoking [4], and smoking-related illnesses, contributing to $300 billion in annual medical costs [5]. Additionally, a national survey in the United States [6] and systematic review of the existing literature [7] highlight that one in four nonsmokers have experienced ETS exposure, with such exposure related to negative health outcomes, including chronic respiratory illness.

One particular chronic respiratory illness that continues to impact 20 million American adults is asthma [8], a chronic disease of the lung characterized by coughing, wheezing, shortness of breath, and tightness of the chest [9,10]. Tobacco smoke is a known trigger for asthma, often due to airway inflammation and corticosteroid resistance [11,12]. Nevertheless, much of the literature focusing on the impact of tobacco and ETS exposure on asthma have addressed prevalence of asthma or asthma onset, primarily among children. In this study, we aimed to expand the empirical evidence and evaluate the independent role of exposure to ETS, as well as tobacco use (cigarette and electronic cigarettes), on asthma severity among adults with current asthma. Further, given calls to address the role of biological sex in health [13], especially related to nicotine [14], we further stratified our analysis by sex. 

## 2. Methods

We conducted a secondary analysis of the California Health Interview Survey (CHIS), the largest state health survey in the United States [15]. 

### 2.1. Study Population

The sample for this study consisted of California adults aged 18 years or more from the latest released CHIS data files (2020). CHIS provide raked survey weights which were used in this study for Jackknife variance estimation in creating population-based estimates for California. For the year 2020, CHIS used a mix of web and telephone-based surveys to contact participants. Further information can be found through the 2019–2020 CHIS Methodology Reports [16]. For this study, we used CHIS-provided variables indicating respondents who noted physician/health care diagnosis of asthma and still having current asthma were included as the sample. 

### 2.2. Measures

The primary outcome of this study was self-reported asthma attack in the past 12 months (yes or no). Smoking status was defined as saying “yes” to current electronic cigarette or cigarette smoking, with non-exposure defined as not a current smoker. ETS exposure was defined when smoking was noted as “allowed in some places or sometime” or “allowed anywhere and anytime” when the respondent was asked “Which statement best describes smoking or vaping a tobacco product, including e-cigarettes, inside your home?” or “yes” to the question “In the last two weeks, have you ever been exposed to secondhand tobacco smoke or e-cigarette vapor in California?”. Additional control variables included were: age (18–25 years, 26–44 years, 45–64 years, 65 years or more), race/ethnicity (Latino, White, African-American, Asian, Other), poverty level (200% federal poverty level [FPL] or more, Less than 200% FPL), highest educational attainment (high school or less; some college, vocational school, or associates degree; bachelor’s degree or higher), health insurance status (insured all past 12 months, not insured all past 12 months), body mass index (BMI) category (dichotomized as normal or underweight, overweight or obese), past year serious psychological distress (SPD) measured using the validated [17] Kessler scale (yes or no), ever received positive test result for COVID-19 (yes or no), and has an asthma management plan from a healthcare professional (yes or no). 

### 2.3. Data Analysis

All data analyses were survey-weighted and conducted using SAS v9.4 (SAS Institute Inc.; Cary, NC, USA). To obtain sociodemographic characteristics and the prevalence of our primary variables of interest, survey-weighted population size and survey-weighted percentage were first obtained by sex assigned at birth, as provided by CHIS (male, female). Next, to evaluate putative association between each population characteristics, including ETS exposure and current smoking status to that of asthma attack, we conducted sex-stratified crosstabulations using survey-weighted Rao-Scott Chi-Square with *p* < 0.05 denoted to assess significance. Survey-weighted percentage calculated from population estimates were calculated and thus presented in bivariate analyses. Next, we conducted sex-stratified survey-weighted binary logistic regression to model the effect of ETS exposure, current smoking status, as well as control variables on asthma attack in the past 12 months, with *p* < 0.05 used to assess significance. Finally, we excluded all participants who reported being current smokers (cigarette or electronic cigarette), and conducted a sub-analysis to evaluate the role of ETS exposure on asthma severity among non-smoking adults with asthma. 

## 3. Results

As shown in Table 1, among the primary variable of interest, 35% of males and 30% of females reported ETS exposure in the past 12 months, while 13% of males and 6% of females reported being a current smoker. Past year asthma attack was reported among 43% and 55% of males and females, respectively. Further, 34% males and 27% of females reported not having an asthma management plan from healthcare facility/professional. Further, in order to provide an understanding of the study population, we present the study population characteristics in Table 1 as well. 

Table 2 provides the results of chi-square test between each study population characteristics and asthma attack, by sex. Males who reported ETS exposure, compared to those who did not, had a significantly higher prevalence of past year asthma attack (56% vs. 37%). Likewise, the prevalence of asthma attack was significantly higher among male current smokers (68%) as compared to male non-current smokers (41%). No such significant association was found among females. All variables noted in Table 2 were further included in regression analyses. 

Table 3 highlights the odds ratios (OR) and 95% confidence intervals (CI) of asthma attack (by sex). Due to the association with additional study population characteristics, we provided the full models. Among males, after adjusting for all control variables (age, race/ethnicity, poverty level, highest educational attainment, insurance status, having an asthma management plan, BMI, SPD, and COVID-19 status), asthma attack was significantly higher among those with ETS exposure (OR: 1.75, 95% CI: 1.01–3.02) and among current smokers (OR: 3.82, 95% CI: 1.49, 9.81). Among females, lower odds of asthma attack was associated with being Asian (OR: 0.49, 95% CI: 0.29, 0.84), while having an asthma management plan was associated with higher odds of asthma attack (OR: 1.51, 95% CI: 1.07, 2.12). All relevant interactions were assessed. 

Among male non-smokers with current asthma, 29% reported ETS exposure. Among female non-smokers with current asthma, 28% reported ETS exposure. In addition, male non-smokers with current asthma, 40% reported asthma attack in the past year. Among female non-smokers with current asthma, 55% reported asthma attack in the past year. As further shown in Table 4a, male non-smokers with current asthma who had ETS exposure, had a significantly higher prevalence of asthma attack (49%), when compared to those without ETS exposure (36%); with no significant association found among females. As shown in Table 4b, upon regression analyses (model adjusted for age, race/ethnicity, poverty, education, insurance, having asthma management plan, psychological distress, and COVID-19 status), male non-smokers with ETS exposure had a 109% higher odds of asthma attack, compared to those without such exposure. Among females, having an asthma management plan continued to yield higher odds of asthma attack as well. 

## 4. Discussion

Our results, using a population-based survey, demonstrate that among adult males with current asthma, being a current smokers and exposure to ETS were both independently related to asthma attack, even after accounting for sociodemographic characteristics, insurance status, psychological distress, obesity, having an asthma management plan, and COVID-19 status. Such results demonstrate the ongoing burden of tobacco use and exposure among chronically ill males and corroborate the literature on tobacco and asthma to some extent; though our results highlight putative sex-specific outcomes. 

For example, in a study in Taiwan, household ETS exposure was shown to increase the prevalence of asthma and related symptoms among children [18], with a similar trend noted among children in the United States [19] and a meta-analysis confirming the modest association between childhood asthma diagnosis and ETS exposure [20]. Further, Jin et al. noted that ETS was associated with increased hospitalization among children with asthma [21], a trend further confirmed by Wang et al. in a systematic review and meta-analysis as well [22]. Likewise, exposure to smoke from electronic nicotine delivery systems has been shown to increase asthma attack among children [23]. Though similar studies among adults are limited, Eisner et al. found that ETS exposure, measured through personal nicotine badge, was associated with asthma severity among adults enrolled in a northern California managed care organization [24]. Our study expands such literature and not only confirms the higher prevalence of asthma attack associated with both firsthand smoke and ETS exposure among adults, but also highlights sex-specific trend in asthma severity upon such exposures, with a disproportionate burden among males.

While laboratory studies and expert reviews note that impact of nicotine on gonadal hormones, as well as the role of sex hormones on inflammatory process of the lung, may contribute to biological-sex based differences in disease progression, and worse outcomes among females [14,25], it does not explain the higher burden noted among males in our study, which could stem from societal masculine roles. For example, the current evidence notes that males report lower prevalence and severity of asthma when compared to females [26], a pattern noted in our study. Yet, as noted in the literature, males are less likely to report poorer health or use preventive services, when compared to females [27,28,29]. It is thus feasible that such lower healthcare utilization may contribute to increased severity of asthma, which is further exacerbated by ETS exposure and smoking behavior. 

Such results call for sex-specific asthma management plans where an assessment and plans to reduce exposures to firsthand and ETS among males would be critical to ensuring improved quality of life. Thus, future studies are needed to assess efficacy of such sex-based management plans. Likewise, the unique association between having an asthma management plan and increased severity of asthma among females warrants further analysis. It is plausible that those with severe asthma and ongoing attacks are more likely to receive such a plan and our cross-sectional analysis do not allow for a temporal assessment of whether the plan was received before or after onset of attacks. Nevertheless, given the portion of females with current asthma that remain without management plans, there is a need for more targeted efforts to ensure asthma management plans, such as the one recommended by the Centers for Disease Control and Prevention [30], for the population. 

Furthermore, the empirical evidence highlights that overweight and obesity status can increase asthma severity, among pediatric and adult populations [31,32], as can the presence of mental illness [33,34]. In our study, we accounted for such factors. However, both firsthand and ETS exposure remained significant among males, thus showing that public health and clinical efforts to optimize control of symptoms among asthmatics with higher body mass index and/or mental illness continue to address smoking behavior as well as efforts to reduce ETS exposure. 

Finally, upon excluding current smokers, the association between ETS exposure and an asthma attack for male asthmatics not only remained significant, but odds of such attack increased by two-fold, highlighting the ongoing burden of ETS exposure and the need for strict tobacco-control policies. While California remains the forefront in tobacco legislation and thus prohibits smoking indoors and in workplaces [35,36], legislations related to ETS exposure multi-unit dwelling units and outdoor shared spaces remain limited. For example, in San Bernardino County within California, which is also largest geographic county in the United States, a Hispanic majority (54.4%), with a Gini index (measure of income inequality) of 0.497 (higher than national average), and 16% poverty rate [37], tobacco control policies remain limited, as demonstrated by 22 out of 25 areas with grade Ds and Fs for smoke-free outdoor air and 24 out of 25 with grades of Fs for smoke-free, as noted by the State of Tobacco Control 2021-California Local Grades report [38], thus highlighting limited tobacco-control policies in disparity areas. Nevertheless, over 230 cities and counties in the state have banned outdoor smoking [39], thereby reducing ETS exposure. Coupled with outlines such as *Smoke-Free Multiunit Housing Model Ordinance* [40], the model to implement state-wide ETS exposure reduction legislation is both imperative and feasible not just within the state, but the nation as well (where tobacco use remains epidemic). 

The results of our study should be interpreted in the context of its limitations. CHIS is a cross-sectional study and thus does not allow for causal or temporal relationships to be evaluated. Future studies should address the time to ETS exposure to asthma attack to better provide a timeline for an asthma management plan. Further, CHIS data is susceptible to recall and self-report bias and given the sensitive topic of tobacco use, some participants may have under-reported their smoking behavior as well. In addition, the CHIS public access data lacks further details on sexual and gender minorities (SGM), and thus were limited to sex assigned at birth stratification only. Due to the overlap of participants who used cigarettes and electronic cigarettes, we did not conduct separate analyses. However, future studies may benefit from providing such disaggregated data. Finally, the most recent CHIS data excluded asthma-related healthcare utilization, and thus the results remain limited to previous year data on such sub-analysis.

Notwithstanding such limitations, the results of our study highlight sex-specific disproportionate burden of firsthand and ETS exposure, independently, among males with current asthma. The results of our study show the need for tailored interventions for males to optimize symptom management through both reduction of active smoking as well as public health efforts to create smoke-free spaces. Further, we used the largest state health survey, CHIS, which includes data from all 58 counties in 44 geographic areas in the State, in multiple languages (such as Cantonese, Mandarin, Vietnamese, Korean, Tagalog, and Spanish), and uses random selection (through landline and cellphone phone numbers) of state residents [15]. Our survey-weighted analysis further reduces threat to external validity and allows for generalizability to the State as well to allow for evidence-based decision making on tobacco control.

## Figures and Tables

**Table 1:** Study population characteristics, by sex (n males = 700, N males = 1,054,927, n females = 1510, N females =1,642,010).

	Males Only N, %	Females Only N, %
Asthma Attack Past Year		
No	596,513 (56.55)	743,505 (45.28)
Yes	458,414 (43.45)	898,504 (54.72)
Has asthma management plan from health professional		
No	363,274 (34.44)	450,144 (27.41)
Yes	691,653 (65.56)	1,191,866 (72.59)
Smoking Status (Cig+Ecig)		
Not Current Smoker	918,010 (87.02)	1,545,648 (94.13)
Current Smoker	136,917 (12.98)	96,361 (5.87)
Secondhand Smoke Exposure (combined)		
No	689,120 (65.32)	1,141,532 (69.52)
Yes	365,807 (34.68)	500,477 (30.48)
Age		
18–25	171,996 (16.30)	213,086 (12.98)
26–44	394,351 (37.38)	442,749 (26.96)
45–64	280,619 (26.60)	577,335 (35.16)
65 or more	207,961 (19.71)	408,839 (24.90)
Race/Ethnicity		
White	420,935 (39.90)	750,128 (45.68)
African-American	80,171 (7.60)	127,976 (7.79)
Latino	185,316 (17.57)	320,892 (19.54)
Asian	82,031 (7.78)	144,624 (8.81)
Other	286,475 (27.16)	298,390 (18.17)
Poverty Status		
200% FPL or more	829,617 (78.64)	1,088,101 (66.27)
Less than 200% FPL	225,311 (21.36)	553,909 (33.73)
Education Status		
High School or Less	323,609 (30.68)	563,883 (34.34)
Some College, Vocational, Associates	267,819 (25.39)	379,322 (23.10)
Bachelors or higher	463,499 (43.94)	698,805 (42.56)
Insurance Status		
Not all insured past 12 months	91,705 (8.69)	126,409 (7.70)
Insured all past 12 months	963,222 (91.31)	1,515,600 (92.30)
BMI Category		
Normal or Underweight	292,491 (27.73)	516,265 (31.44)
Overweight or Obese	762,437 (72.27)	1,125,745 (68.56)
Past Year Psychological Distress		
No	841,396 (79.76)	1,318,239 (80.28)
Yes	213,532 (20.24)	323,771 (19.72)

**Table 2:** Association between study population characteristics and asthma attack in the past 12 months, by sex.

Variables	Males with Asthma Attack	Females with Asthma Attack
Age		***
18–25	34.61	49.44
26–44	44.10	59.45
45–64	47.23	63.97
65 or more	44.45	39.29
Race/Ethnicity		
White	47.80	56.46
African-American	41.39	62.16
Latino	31.58	50.62
Asian	39.07	41.17
Other	46.59	58.13
Poverty Status	*	
200% FPL or more	40.48	56.19
Less than 200% FPL	54.42	51.83
Education Status		*
High School or Less	43.26	47.84
Some College, Vocational, Associates	40.93	58.39
Bachelors or higher	45.05	58.28
Insurance Status		
Not all insured past 12 months	59.56	61.70
Insured all past 12 months	41.92	54.14
BMI Category		
Normal or Underweight	38.14	52.57
Overweight or Obese	45.49	55.71
Smoking Status (cig ecig)	**	
Not Current Smoker	40.67	54.96
Current Smoker	67.34	50.07
Secondhand Smoke Exposure (combined)	**	
No	37.00	53.27
Yes	55.61	58.04
Past Year Psychological Distress		
No	41.63	53.61
Yes	50.64	59.22
Has asthma management plan from healthcare professional		
No	41.22	48.64
Yes	44.63	57.02

* *p* < 0.05, ** *p* < 0.01, *** *p* < 0.001.

**Table 3:** Odds ratio (OR) and 95% confidence interval (CI) of asthma attack in the past 12 months, by sex ^†^.

Variables	Males OR (95%CI)	Females OR (95%CI)
Age		
18–25	Ref.	Ref.
26–44	1.47 (0.65, 3.32)	1.44 (0.69, 3.00)
45–64	2.15 (0.92, 5.00)	1.73 (0.87, 3.46)
65 or more	2.13 (0.71, 6.41)	0.66 (0.34, 1.28)
Race/Ethnicity		
White	Ref.	Ref.
African-American	0.70 (0.30, 1.60)	1.15 (0.64, 2.07)
Latino	0.65 (0.27, 1.56)	0.78 (0.43, 1.39)
Asian	0.94 (0.43, 2.06)	0.49 (0.29, 0.84) **
Other	0.90 (0.42, 1.93)	0.95 (0.59, 1.56)
Poverty Status		
200% FPL or more	Ref.	Ref.
Less than 200% FPL	1.78 (0.91, 3.47)	0.98 (0.65, 1.46)
Education Status		
High School or Less	0.63 (0.33, 1.21)	0.75 (0.45, 1.25)
Some College, Vocational, Associates	0.69 (0.35, 1.35)	1.11 (0.79, 1.57)
Bachelors or higher	Ref.	Ref.
Insurance Status		
Not all insured past 12 months	2.23 (0.71, 6.99)	1.40 (0.59, 3.31)
Insured all past 12 months	Ref.	Ref.
BMI Category		
Normal or Underweight	Ref.	Ref.
Overweight or Obese	1.57 (0.85, 2.90)	1.19 (0.87, 1.63)
Smoking Status (ecig and cig)		
Not Current Smoker	Ref.	Ref.
Current Smoker	3.82 (1.49, 9.81) **	1.17 (0.84, 1.63)
Secondhand Smoke Exposure (combined)		
No	Ref.	Ref.
Yes	1.75 (1.01, 3.02) *	0.63 (0.35, 1.15)
Past Year Psychological Distress		
No	Ref.	Ref.
Yes	1.72 (0.93, 3.17)	1.20 (0.76, 1.90)
Has asthma management plan from healthcare professional		
No	Ref.	Ref.
Yes	1.51 (0.90, 2.54)	1.51 (1.07, 2.12) *

* *p* < 0.05, ** *p* < 0.01. ^†^ Logistic regression models adjusted for self-reported COVID-19 positive status.

**Table 4:** a: Prevalence of asthma attack in the past 12 months, by sex, among non-smokers with current asthma. b: Odds ratio (OR) and 95% confidence interval (CI) of asthma attack in the past 12 months, by sex, among non-smokers with current asthma.

(**a**)		
**Variables**	**Males** **%**	**Females** **%**
Secondhand Smoke Exposure (combined)	*	
No	35.59	53.71
Yes	49.18	58.71
Has asthma management plan from healthcare professional		*
No	38.69	48.12
Yes	39.99	57.77
(**b**)		
**Variables**	**Males** **OR (95%CI)**	**Females** **OR (95%CI)**
Secondhand Smoke Exposure (combined)		
No	Ref.	Ref.
Yes	2.09 (1.19, 3.67) *	1.11 (0.77, 1.62)
Has asthma management plan from healthcare professional		
No	Ref.	Ref.
Yes	1.31 (0.72, 2.36)	1.56 (1.10, 2.22) *

Model further adjusted for age, race/ethnicity, poverty level, highest educational attainment, insurance status, BMI, SPD, and COVID-19 status. * *p* < 0.05

## Data Availability

Publicly available dataset was analyzed in this study. This data can be found here: https://healthpolicy.ucla.edu/chis/data/Pages/GetCHISData.aspx (accessed on 18 February 2022).
